# Characterization and Separation Performance of a Novel Polyethersulfone Membrane Blended with Acacia Gum

**DOI:** 10.1038/s41598-017-14735-9

**Published:** 2017-11-20

**Authors:** Yehia Manawi, Viktor Kochkodan, Ebrahim Mahmoudi, Daniel J. Johnson, Abdul Wahab Mohammad, Muataz Ali Atieh

**Affiliations:** 1 Qatar Environment and Energy Research Institute (QEERI), Hamad bin Khalifa University (HBKU), Qatar Foundation, Doha, Qatar; 2 College of Science and Engineering, Hamad Bin Khalifa University, Qatar Foundation, PO Box, 5825 Doha, Qatar; 3 Department of Chemical and Process Engineering, Faculty of Engineering and Built Environment, Universiti Kebangsaan Malaysia, 43600 Bangi, Selangor Darul Ehsan Malaysia; 4 College of Engineering, Swansea University, Bay campus Swansea, SA1 8PP Wales, United Kingdom

## Abstract

Novel polyethersulfone (PES) membranes blended with 0.1–3.0 wt. % of Acacia gum (AG) as a pore-former and antifouling agent were fabricated using phase inversion technique. The effect of AG on the pore-size, porosity, surface morphology, surface charge, hydrophilicity, and mechanical properties of PES/AG membranes was studied by scanning electron microscopy (SEM), Raman spectroscopy, contact angle and zeta potential measurements. The antifouling -properties of PES/AG membranes were evaluated using *Escherichia coli* bacteria and bovine serum albumine (BSA). The use of AG as an additive to PES membranes was found to increase the surface charge, hydrophilicity (by 20%), porosity (by 77%) and permeate flux (by about 130%). Moreover, PES/AG membranes demonstrated higher antifouling and tensile stress (by 31%) when compared to pure PES membranes. It was shown that the prepared PES/AG membranes efficiently removed lead ions from aqueous solutions. Both the sieving mechanism of the membrane and chelation of lead with AG macromolecules incorporated in the membrane matrix contributed to lead removal. The obtained results indicated that AG can be used as a novel pore-former, hydrophilizing and antifouling agent, as well as an enhancer to the mechanical and rejection properties of the PES membranes.

## Introduction

Polyethersulfone (PES) is a low price commercial polymer, which is widely used in phase inversion fabrication of the polymer membranes for microfiltration, ultrafiltration and gas separation due to its high mechanical strength and chemical stability^1^. However, PES is quite hydrophobic material (water contact angle between 65 and 80 degrees^2^) and membrane fouling is one of the main drawbacks of PES membranes^3,4^. As membrane fouling is more severe with hydrophobic membranes due to the hydrophobic interactions between solutes and the membranes^5^, the increase in the hydrophilicity of PES membranes is considered as one of the key approaches to mitigate this undesirable phenomenon. Whereas some researchers tried to change the surface properties of PES membrane to increase the hydrophilicity^6,7^, other researchers used some additives to the cast solution for the sake of lowering the membrane hydrophobicity and enhancing the membrane flux. Some of the frequently used hydrophilic non-solvent additives are: polyvinylpyrrolidone (PVP)^8,9^, alcohols^10^, polyethylene glycol (PEG)^11,12^ and cellulose acetate phthalate^13^. Such hydrophilic additives are known to cause thermodynamic instability and enhance the instantaneous de-mixing in the casting solutions; as a result, more hydrophilic membranes with larger pore size and higher flux are formed^9^. Moreover, amphiphilic compounds, which contain both hydrophobic and hydrophilic segments were also used as additives during casting of PES membranes^14^. Loh *et al*.^15^ found that hydrophilic segments of the amphiphilic additives segregated to the polymer/water interface, with the hydrophobic segment firmly anchored in the polymer matrix^4,15^. The addition of amphiphilic soybean phosphatidylcholine to the casting solution was reported to increase the membrane skin layer thickness and makes it more hydrophilic^16^. Amphiphilic sulfobetaine copolymer was added to a dope solution to increase hydrophilicity of PES membranes^17^. The flux recovery ratio of the membrane containing 1.57 wt.% of the copolymer in the casting solution was found to increase by more than 40%^4^. More details on using of amphiphilic additives for casting PES membranes might be found in a recent review^4^.

Lately, Acacia gum (AG) has received some significant attention by many scientists due to its amphiphilic nature^18–21^. AG is a natural gum which is extracted from the dried gummy exudation which can be found in some acacia trees species^22^. AG is mainly composed of high molecular weight polysaccharides (∼97%) and a proteionuos fraction (∼3%)^23^. The main mono-saccharides in AG are arobinopyranose (Ara), glucuronic acid (Glca), galactose (Gal) and rhamnose (Rha), while the main amino acids are alanine, arginine, glutamic acid, glycine and histidine^23^. AG is widely used in food industry as a surfactant and emulsifier^24^. Moreover, AG is extensively used in cosmetics, pharmaceutics and dentistry to combat periodontic bacteria and deposition of plaque due to its anti-bacterial properties^25^. Interestingly, AG is extensively used in the Middle East as a chewing gum to beat the harmful bacteria in the mouth which cause several gum diseases. Gazi *et al*.^26^ have found that chewing gums made with AG are more efficient in reducing plaque after seven days when compared with regular sugar-free gums. These amphiphilic and antibacterial properties of AG as an additive during membrane casting might be promising for improvement of hydrophilic and the antifouling properties of the PES membranes.

However, up to now, application of AG in the fabrication of PES membranes has not been reported in literature. In this study, AG for the first time has been used as an additive during casting of PES membranes. The aim of this work is to investigate the effect of using AG as an additive to casting solution on the properties and performance of PES membranes including its effect on pore size, porosity, flux, hydrophilicity, surface charge, antifouling and mechanical properties. The prepared PES/AG membranes were also used for lead removal from aqueous solutions. Lead is one of the heavy metals which have been strongly related to numerous health problems due to its high toxicity which can cause permanent intellectual disability and cancer^27^. Since lead was considered as persistent, bioaccumulative and toxic (PBT), it was banned from use in paints (in 1978) and fuels (in 1996) and now is replaced with other material which has less impact on environment^28^. However, as being persistent, lead is still found at above EPA action level of 15 ppb^29^ in surface waters and groundwater in many parts of the world^28^. Therefore, treatment of lead in water is imperative to circumvent human health risks.

## Materials and Methods

### Materials

N-Methyl-2-pyrrolidone (NMP), AG with a molecular weight of approximately 250,000 and bovine serum albumin (BSA) (molecular weight of 69 KDa) were purchased from Sigma-Aldrich (USA). PES (Ultrason-E 3010 in pellet form) was purchased from BASF (Germany). The non-solvent used in the coagulation bath was Millipore deionized water (DW).

### Preparation of PES/AG membranes

PES/AG membranes were cast using the phase inversion technique using PHILOS flat sheet membrane casting system (Republic of Korea). PES/AG membrane samples were prepared with different loadings of AG (0.3, 0.5, 1.0, 1.5 and 3.0 wt.%) in the dope 16 wt.% PES/NMP solutions following the procedure described earlier^30^. For the preparation of the casting solutions; NMP, PES pellets and AG were mixed using digital mechanical stirrer (an IKA Ultra Turrax T25, USA) for 2 h at 60 °C. The dope solution was then degassed for about 6 h and cast on the surface of a clean glass plate using a casting knife with a gap height of 200 µm at casting speed of 2.5 m/min at 25 °C. After casting, the glass plate with a PES film was immersed in DW at room temperature (21 °C) and kept until the membrane was completely peeled off from the plate. The cast membranes were then washed and kept in DW for 24 h at room temperature to remove any traces of the solvent.

The dynamic viscosity of the casting solutions at different loading of AG was measured using an LVDVE115 viscosimeter (Brookfield Inc, USA). At least three measurements have been performed for each casting solution.

### Membrane characterization and testing

#### Surface morphology and porous structure

The top and cross-sectional structures of the fabricated membranes were analyzed using Field Emission Scanning Electron Microscopy (FESEM) (Gemini model SUPRA 55VP-ZEISS) equipped with Energy-dispersive X-ray spectroscopy (EDX) (Oberkochen, Germany). Membrane samples were fractured using liquid nitrogen and all the membrane samples were coated with platinum before scanning. Vacuum condition (at 3 kV) was applied in order to obtain high resolution surface and cross-sectional SEM images of the membrane.

Gravimetric method was used to evaluate the porosity of the membrane^31^. In this method, the membranes were dried by keeping them inside an oven at a temperature of 50 °C for 24 h before weighing their mass (*w*_*d*_). After that, the dried membranes were immersed in distilled water at 25 °C for another 24 h. The water droplets on the membranes surface were carefully wiped off using filter paper when the membrane was removed from water in order to be weighed again (*w*_*w*_). Equation (1) is used to figure out the total porosity of the membrane (*ε*) using the average values of the wet and dry masses of each membrane sample:

1 $$\varepsilon =\,\frac{{w}_{w}-{w}_{d}}{A.l.\rho }\times 100$$

where *w*_*w*_, *w*_*d*_ are the wet and dry masses of the membranes, respectively, *ρ* is the density of the distillated water at 25 °C^31^, A is the membrane surface area (m^2^) and l is the thickness of the membranes.

The pore sizes of the prepared membranes can be figured out using Guerout–Elford–Ferry equation and the membrane porosity values^32^:

2 $${r}_{m}=\sqrt{\frac{(2.9-1.75\varepsilon )8\,\eta lQ}{\varepsilon A{\rm{\Delta }}P}}$$

where *Q* is the volume of pure water (permeate) expressed in m^3^/s, *η* is the viscosity of water at 25 °C which is 8.9 × 10^−4^ Pa s and *ΔP* is the operating pressure which is 4 bar.

#### Membrane hydrophilicity and surface charge

Ramé-hart Model 200 standard contact angle goniometer was used to measure the water contact angle between the water droplet and the membrane surface with a drop size of 2.0 μl. The contact angle of every sample was measured at 3 different points on the membrane surface.

The electrokinetic analyser SurPass 3 (Anton Paar KG, Austria) was used to measure the zeta potentials of the membrane surfaces. Helmholz–Smoluchowsky equation was used to figure out the value of the zeta potential from the slope of the streaming potential versus operating pressure plot (Equation 3)^33^:

3 $${\rm{\zeta }}=\frac{{\rm{\Delta }}E\,{\rm{\mu }}\,k}{{\rm{\Delta }}P\,{{\rm{\phi }}}_{0}{{\rm{\phi }}}_{{\rm{r}}}}$$

where *k* is the conductivity of the electrolyte, *μ* is the solution viscosity, *ΔE* is the streaming potential, *φ*_*r*_ is the dielectric constant of water (at 25 °C), *φ*_0_ is the vacuum permittivity of a and *ΔP* is the pressure drop across the membrane. Surpass 3 electrokinetic analyser can be described simply as a measuring cell in which two membrane pieces are positioned facing each other with 100 µm gap in between and an aqueous solution of 1 mM KCl and pH of 5.3 ± 0.2 passes inside the measuring cell. Two silver electrodes were employed to figure out the electrical potential difference. In order to conduct a pH scan of the zeta potential (zeta potential measurement at different pH values), 0.1 M NaOH and 0.1 M HCl solutions were used to change the pH of the KCl solution.

#### Fourier Transform Infrared (FTIR) and Raman spectrometer

Nicolet 6700 Thermo Scientific-FITR spectrometer (USA) and Thermo Scientific Nicolet Raman spectrometer were used to analyze the FTIR and Raman spectra of the membrane samples.

#### Membrane filtration tests

A dead-end stirred cell (HP4750X from Sterlitech (USA)) was used to carry out the filtration tests. The used cell has a volume of 0.3 L and the membrane area of 14.6 cm^2^. Nitrogen gas was used for the pressurization of the cell.

Equation (4) is used to figure out the amount of permeate flux (J) obtained:

4 $$J=\frac{Q}{A.T}$$

where *A* is the membrane cross-sectional area (m^2^), *T* is the time of permeation (hours), and *Q* is the permeate volume (L).

Equation (5) was used to estimate the lead rejection with the membrane sample:

5 $$R( \% )=(1-\frac{{C}_{P}}{{C}_{f}})\times 100$$

where *C*_*f*_ is the concentration of lead in the feed and *C*_*p*_ is the concentration of lead in the permeate solution.

Lead solutions of 500 ppb were prepared from lead nitrate (PbNO_3_) and used as a feed. Aurora Elite-Bruker Inductively coupled plasma mass spectrometry (Germany) was used to evaluate the lead concentration in the feed and permeate. Bovine serum albumin (BSA) solution at a concentration of 100 mg/L and at pH 6.5 was used to conduct the fouling tests of the membranes. In this test, initial deionized water (DW) flux of the membranes (J_0_) was calculated first by filtration of DW for 15 min at operating pressure of 4 bar. After that, the 100 mg/L BSA solution was filtered through membranes for two hours at the same pressure. The membranes were then rinsed thoroughly with DW and water flux after BSA filtration (J_f_) was measured. The normalized flux (J_n_) values were then calculated using equation (6):

6 $${J}_{n}=\frac{{J}_{f}}{{J}_{{\rm{o}}}}\,$$

#### Stability tests of PES/AG membranes

The stability of PES/AG membranes was tested using two different methods; the first method involved FTIR analysing of permeate collected during DW filtration for 3 and 30 min. The spectra were then compared with that of pure DW. The second method involved the immersion of the membrane in DW and sonication with an ultrasonic bath (Branson 8510, USA) with 50/60 Hz for 30 min. After the ultra-sonication, FTIR spectra of the immersing water were recorded and compared with that of pure DW. Also, DW was filtered through the ultra-sonicated PES/AG membranes and the FTIR spectra of the collected permeate were compared with that of DI water.

#### Testing of PES/AG membranes with bacterial suspensions

Escherichia coli *(E.coli*) were used to test the antibacterial behavior of the fabricated membranes. A stock *E.coli* suspension was cultured in nutrient broth media. In this work, an *E.coli* suspension with cell concentration of 20 × 10^7^ CFU/ml was prepared from the stock suspension by appropriate dilution in nutrient broth (the dilution is based on optical density of the suspensions at 600 nm) and used for membrane testing. The fabricated membranes were dipped in the *E.coli* suspension for about 10 min before being transferred to be placed on top of nutrient agar plates. The agar plates were inserted into an incubator at 35 °C and left for overnight. FE-SEM was used to inspect the presence of CFUs on the surface of the membranes.

#### Mechanical testing of the membranes

CT3 Texture Analyzer from Brookfield Engineering (USA) was used to analyse the mechanical properties of the membranes. In this test, the uniaxial tensile behaviour of the membranes was analyzed. In order to conduct the test, the fabricated membranes were cut to a rectangular shape with a size of 75 mm and 25 mm before being dried in oven for overnight. The texture analyser is used to determine the peak load at a crosshead speed of 5 mm/min (following ISO 527-3). The analysis was conducted three times for each sample and the average value was reported. As an indication of the mechanical strength of the membranes, the tensile stress values of the membranes were evaluated. Tensile stress values were calculated from the peak load and the cross-section area of the membrane.

## Results and Discussions

### Membrane morphology and pore size

**Figure 1:**
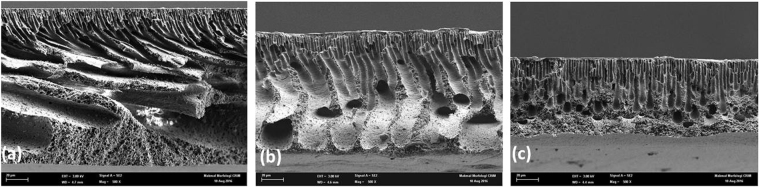
SEM cross-sections of PES membranes at different AG loadings (wt.%) in the dope solutions: (**a**) 0% AG, (**b**) 1% AG and (**c**) 3% AG.

FE-SEM was used to inspect the top and cross-section area of the fabricated membranes. As seen in Fig. 1, both pure PES and PES/AG membranes have a thin top layer and a sponge-like substructure with macro-voids; however, the macro-voids in the PES/AG membrane samples were obviously larger (Fig. 1b and c). Usually, the formation of such types of porous membrane structures is a result of the instantaneous de-mixing during the phase inversion^34^. By enhancing the thermodynamic imbalance in the doping solution, AG behaves as a non-solvent agent in the de-mixing step due to high solubility in water. The overall result is an increase in the phase separation and a faster exchange between non-solvent and the solvent during the de-mixing step which in turn results in increasing the porosity of the top skin layer and the lower sub-layer. In this sense the role of AG in the formation of a porous structure of PES membranes during casting is similar to other polymer additives reported in the literature^35–39^. However as seen in Fig. 1c, high AG loading resulted in decreasing the macro-voids size in the fabricated membrane. This might be explained due to the increase in the viscosity of the casting solutions which slows down the precipitation process and as a result a membrane with lower porosity is formed^40,41^.

**Figure 2:**
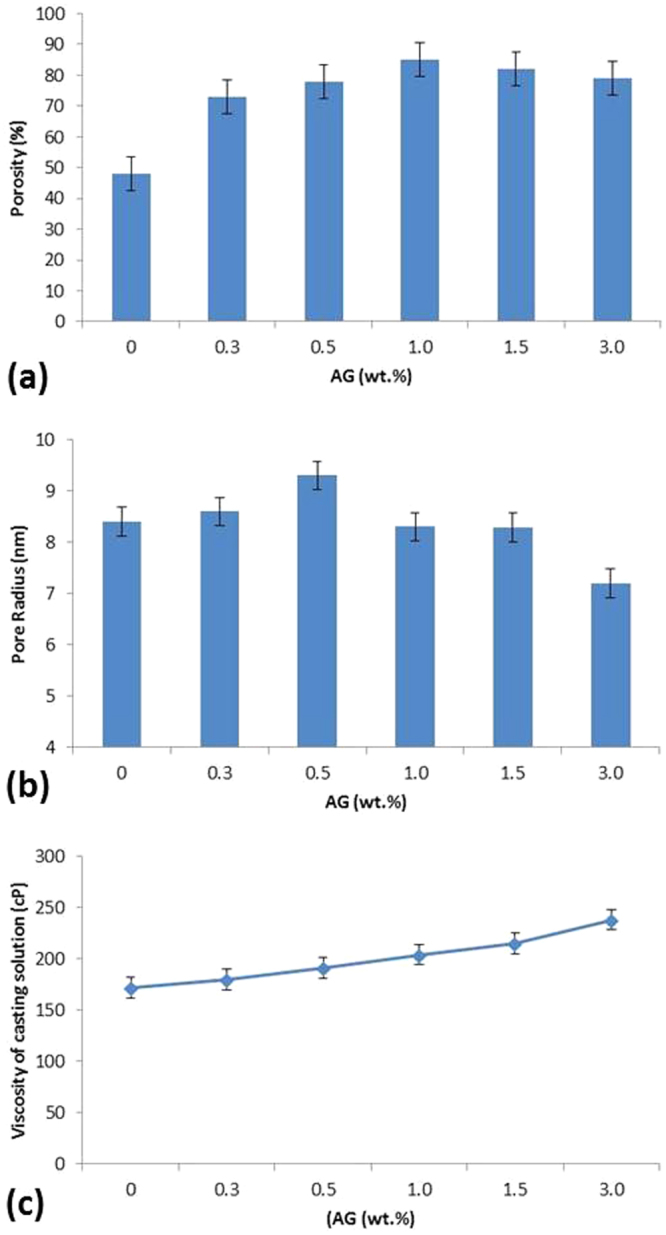
Total porosity (**a**), average pore size (**b**) of PES/AG membranes as well as the dope viscosity (**c**) at different AG loading (wt.%) in the casting solutions.

The SEM findings correlate with data on average porosity of the prepared membrane samples and on viscosity of the casting solutions (Fig. 2). As seen in Fig. 2a, the porosity of the membranes was found to increase with increasing AG content reaching the highest value at 1 wt.% AG in the dope solutions then decreasing slightly after that. The porosity of the PES/AG membrane was found to increase by 77% (at 1 wt.% AG) compared to pure PES membranes. The increase in porosity of the cast membranes when hydrophilic additives were added was also described by Liu *et al*.^40^ and van de Witte *et al*.^41^ who reported that the use of hydrophilic species in the casting solution results in an accelerated solvent and non-solvent exchange which in turn increases the development of a highly porous membrane structure. The additional increase in the AG loading (>1 wt.%) resulted in decreasing the porosity of the fabricated membranes. This might be explained due to the increase in the viscosity of the casting solutions which slows down the precipitation process. The overall effect is a membrane with a thicker top layer with lower porosity and pore interconnectivity^40,41^.

Figure 2b shows the average pore sizes of the fabricated membranes which were calculated using Eq. 2. The average pore radius was between 7.2 and 9.3 nanometers which is in the range of nanofiltration membranes. During the phase inversion process, AG behaved as a non-solvent agent by amplifying the thermodynamic instability in the casting solution. This results in the formation of more porous membrane structure in the presence of AG due to the quick exchange between the solvent and non-solvent. On the other hand, when AG loading in the casting solution was increased beyond 0.5 wt.%, the pore size was found to drop slightly down to 7.2 nm. This behavior has been reported to occur due to impact of high loading of the additive on the viscosity of the casting solutions^42^. At some point the addition of high molecular weight AG increased the viscosity of the casting solutions to such level that the de-mixing ratio between the polymeric and aqueous phases decreased and the membranes with smaller pores were formed.

The measured viscosity data was in good correlation with the above made consumptions. As seen in Fig. 2c, the dynamic viscosity of the casting solutions increases with AG loading from 172 ± 5 cP for the dope solution without AG up to 238 ± 8 cP for the dope solution with 3 wt.% of AG. As the low ratio of hydrophilic AG was added to casting solution, the thermodynamic stability of the casting solution obviously decreased; such phenomena caused an increase in the phase inversion kinetics during the membrane formation. As the mass transfer rate between the solvent (NMP) and nonsolvent (water) increased, we observed formation of membranes with higher porosity and pore size. However, according to Fig. 2c, when 1wt. % or higher of AG was added to the casting solution, the viscosity of the casting solution increased dramatically (204 ± 5 to 238 ± 8 cP). Such increment resulted in lower mass transfer rate between the solvent and non-solvent and membranes with lower porosity and pore size were formed.

### Raman spectra of PES/AG membranes

**Figure 3:**
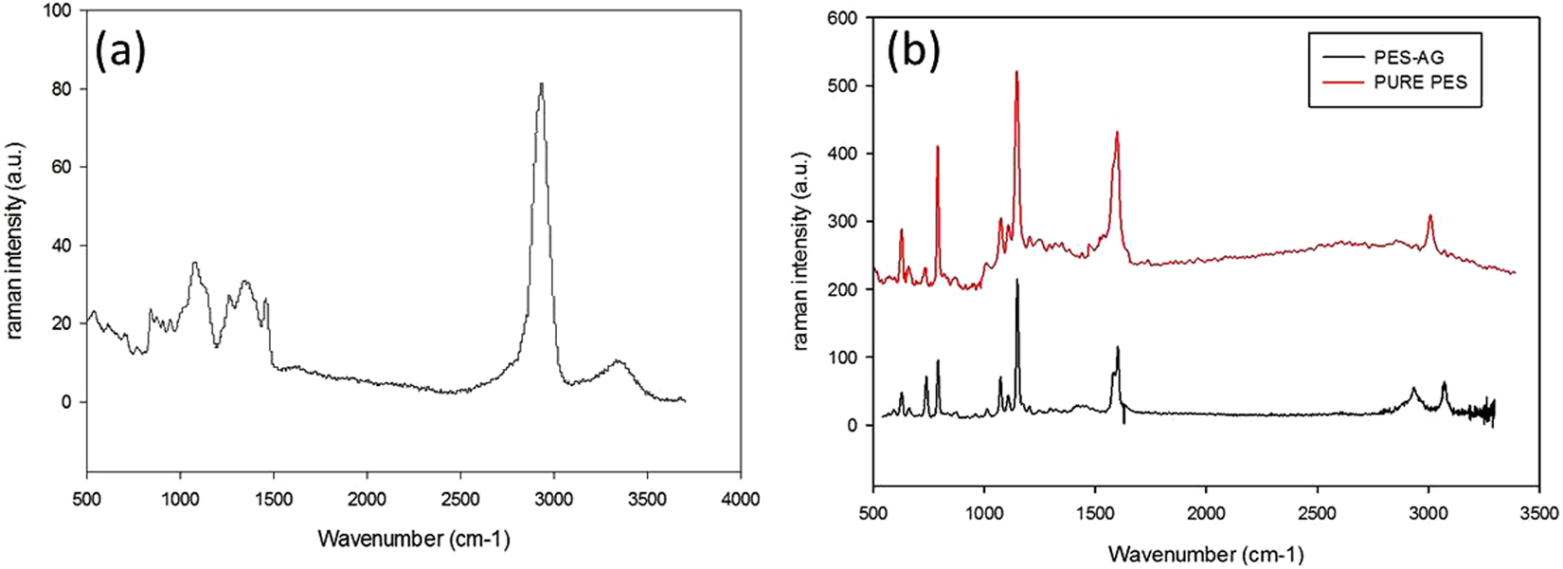
Raman spectra of (**a**) AG and (**b**) pure PES and PES/AG (3 wt.% in the casting solution) membranes.

Figure 3a shows the Raman spectra of AG (powder) while Fig. 3b presents the Raman spectra of the pure PES membrane and PES membrane embedded with AG (3 wt.%). The spectra obtained through Raman analysis confirmed the molecular structure of PES^43^. AG embedded membrane shows a peak at 2852.6 to 2986.4 cm^−1^ which correspond to methylene groups in AG macromolecules. This peak was the most intense Raman peak of pure AG powder as shown in Fig. 3a. The frequency bands of PES and PES/AG show some slight shifting (between 3–6 cm^−1^) after embedding with AG.

### Contact angle and zeta potential measurements

**Figure 4:**
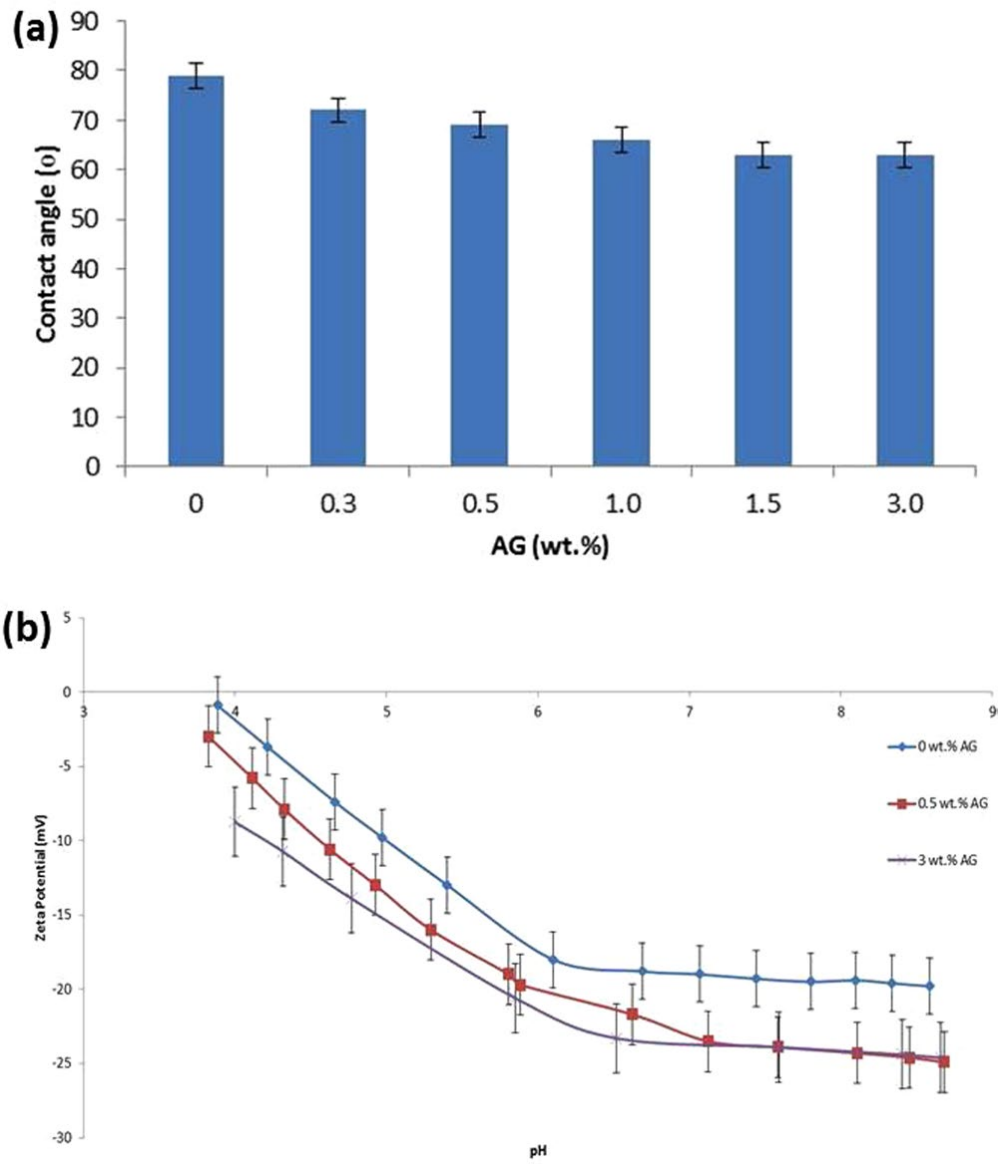
Contact angle (**a**) and zeta potential (**b**) of PES/AG membranes at different AG loading in the casting solutions.

The hydrophilicity of the fabricated membranes was evaluated by measuring the contact angle between the membrane surfaces and water droplets. Figure 4a shows the water contact angles values of PES/AG membranes with different AG loading. The effect of adding AG was found to increase the hydrophilicity of the fabricated membranes by lowering the contact angle by 20% (the sample cast at 3 wt.% AG) when compared to pure PES membrane. The reason behind the drop in the contact angle of PES membranes when AG was added can be explained by the distribution of the AG macromolecules to hydrophilize the membrane surface and pores. This is similar to how the amphiphilic nature of AG is used for the stabilization of oil-water emulsions by the adsorption of the hydrophobic sites of AG macromolecules onto the oil droplets surface whereas the hydrophilic residues of AG macromolecule inhibits droplet aggregation and coalescence^44^. While casting PES/AG membranes, the hydrophobic residues of AG macromolecules possess high interaction with the hydrophobic methyl groups in PES whereas polysaccharide fragments in AG hydrophilize the membrane pores and surface.

Zeta potential of pure PES and PES/AG membranes (0.5, 1.0 and 3.0 wt.%) at different pH values (3.9–8.5) is depicted in Fig. 4b. As shown, pure PES membranes, which were in direct contact with KCl solution, demonstrated relatively small negative zeta potential values between −0.9 and −19.8 mV with marginal increase in charge between pH 6.1 and 8.6. The reason behind the increase in the surface charge of the membrane along with the increase in the pH is explained by Ariza and Benavente^45^ who related this behavior to adsorption of chloride ions from the background electrolyte solution on the membrane surface. The use of AG as an additive to the casting solutions was found to increase the value of the negative zeta potential of the fabricated membranes. The reason behind this could be due to the affixation of some AG macromolecules to PES membranes which end up with (i) strong anchoring of some hydrophobic fragments of AG to PES polymer matrix and (ii) functionalization of the membrane surface with carboxylic groups due to the presence of some polysaccharide residues; these carboxylic groups is thought to dissociate at specific pH conditions. Niu *et al*.^46^ have reported that at pH above 1.9, AG macromolecules act as a weak polyelectrolyte bearing negative charge due to the dissociation of carboxyl groups. The effect of having high surface charge and hydrophilicity on PES membranes is thought to be very useful in lowering the membrane fouling with hydrophobic and charge foulants while separating proteins, bacteria and other organic compounds.

### Water fluxes and lead removal with PES/AG membranes

**Figure 5:**
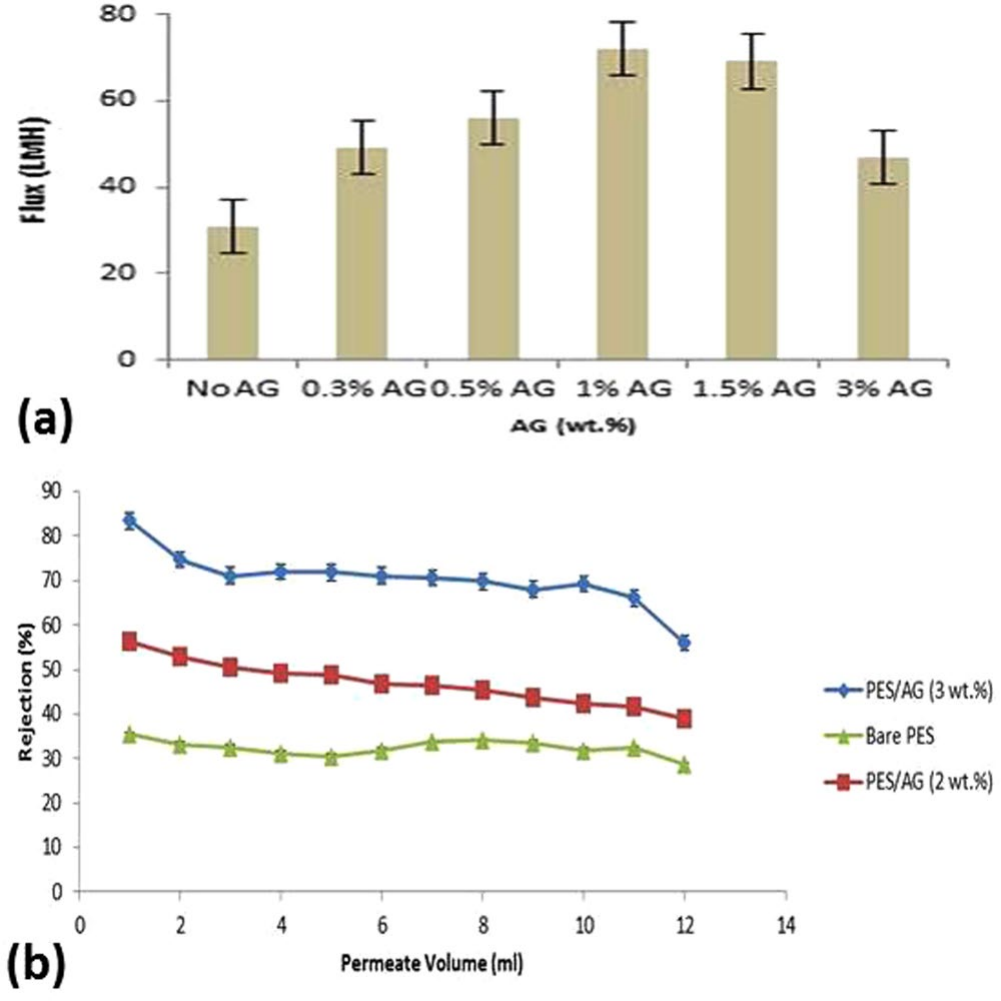
Water fluxes for PES membranes with different AG loading (**a**) and lead rejection versus permeate volume during filtration of 500 ppb lead solutions with bare PES and PES/AG (2 & 3 wt.%) membranes (**b**). pH is 6.5. Operating pressure is 4 bar.

The water flux values of the fabricated membranes at different AG loading in the dope solutions shown in Fig. 5a. The addition of AG into the casting solutions of PES was observed to increase the permeate flux by up to 130% (PES/1 wt.% AG membrane) when compared with pure PES membrane. The flux increase can be attributed to the increase in the membranes’ porosity and hydrophilicity. It should be noted that the flux increase for PES/AG membranes was significantly higher than what has been reported in literature when other additives such as PVP, lithium salts, montmorillonite and 1-vinylpyrrolidone-co-styrene copolymers were used during casting of PES membranes^6,47–49^.

The separation properties of the fabricated membranes were tested by filtration of 500 ppb lead solutions at different feed pH values. As seen in Fig. 5b, lead rejection with PES/2 &3 wt.% AG membranes were much higher than that of pure PES membrane. The rejection capability of PES/AG membranes is thought to occur due to both the sieving (steric) rejection and complexation of lead ions with amino groups of proteionuos fraction of AG. The latter can be justified by conducting energy-dispersive X-Ray spectroscopy (EDS) on the membranes before and after lead filtration. The membrane’s surfaces were analyzed in order to account for the entrapped lead ions within the membrane matrix. Elemental compositions (mas. %) of lead in PES membranes at different AG loading in the dope solutions before and after lead filtrations are shown in Table 1.

**Table 1:** Elemental composition (mas. %) of lead in PES membranes at different AG loading in the dope solutions before and after lead filtration.

Before filtration			After filtration		
Pure PES	PES/1% AG	PES/3%AG	Pure PES	PES/1% AG	PES/3%AG
—	—	—	6.05 ± 1.59	8.17 ± 1.98	13.35 ± 3.12

As seen in Table 1, the percentage of lead bound to PES/AG membrane was higher than that for pure PES membrane. Furthermore, the lead content in PES/3 wt.% AG membrane was higher than that of PES/1 wt.% AG membrane due to a large number of chelating sites available for binding lead ions.

Proteionous fraction of AG contains different amino acids which have strong affinity and distinctive kinetics towards chelating of metallic ions. Amino acids are considered as natural ligands and the ability of amino acids to form lead/amino acid complexes has been widely reported in literature^50–53^. In fact the strong affinity between the metal and amino acids is responsible for the spread and accumulation of metals inside organs which leads to the development of some histological abnormalities in patients under parental nutrition treatment^50,54–57^. Nascimento *et al*.^50^ have listed the order of affinity of amino acids towards lead and cadmium based on the stability constants of metal complexes: alanine> aspartic acid> glutamic acid> glycine> histidine> methionine> phenylalanine> seine> threonine^50^. The presence of these amino acids in significant proportions (as high as 134 residues per 1000 residues of proteinaceous component of commercially-available AG^58^) could be responsible for lead binding with PES/AG membranes. The schematic presentation of lead complexation with glycine present in AG is depicted as an insert in Fig. 5b.

### Antibacterial testing of the fabricated membranes with *E. coli* bacteria

**Figure 6:**
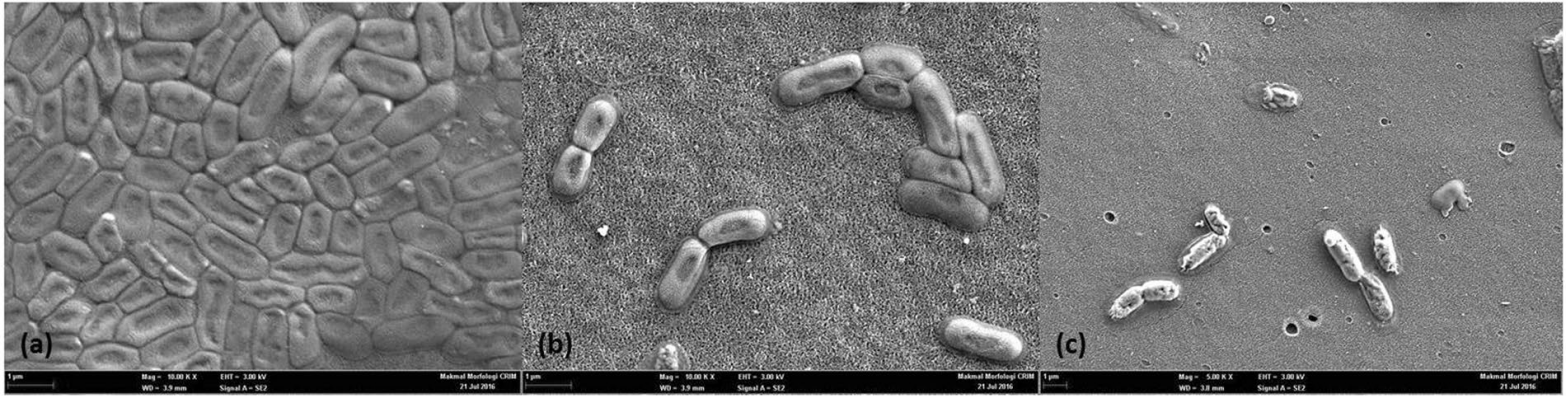
FESEM images of the membrane surfaces after the incubation of *E.coli* cells as described in section 2.3.7: (**a**) Pure PES membrane (**b**) PES membrane with 3 wt. % AG in the dope solution; (**c**) PES membrane with 1 wt. % of AG in the dope solution after filtration of DW for 4 h and incubation of *E.coli* bacteria.

The antibacterial properties of PES/AG membranes were tested by the incubation of *E.coli* on the surface of the fabricated membranes for 24 h. As shown in Fig. 6a, PES membrane surface is thoroughly covered with a layer of bacterial cells after incubation of *E.coli* bacteria. On the other hand, at the identical conditions only few bacterial cells were found on the surface of the PES/3% AG membrane (Fig. 6b). Several research papers have tested and reported the antibacterial properties of AG against different strains of bacteria^59–62^; however, the antimicrobial mechanism of AG has not been covered fully in literature. Tyler^63^ and Kirtikar^64^ reported that the reason behind the antibacterial properties of AG could be due to the presence of some active enzymes (such as: peroxidases, oxidases and pectinases) which are known to have antimicrobial properties^65^. In addition to the presence of these antibacterial enzymes, we believe that the antifouling properties of PES/AG membranes could also be due to the increase in hydrophilicity and surface charge which has an effect on lowering the attachment of bacterial cells on the membrane surface.

**Figure 7:**
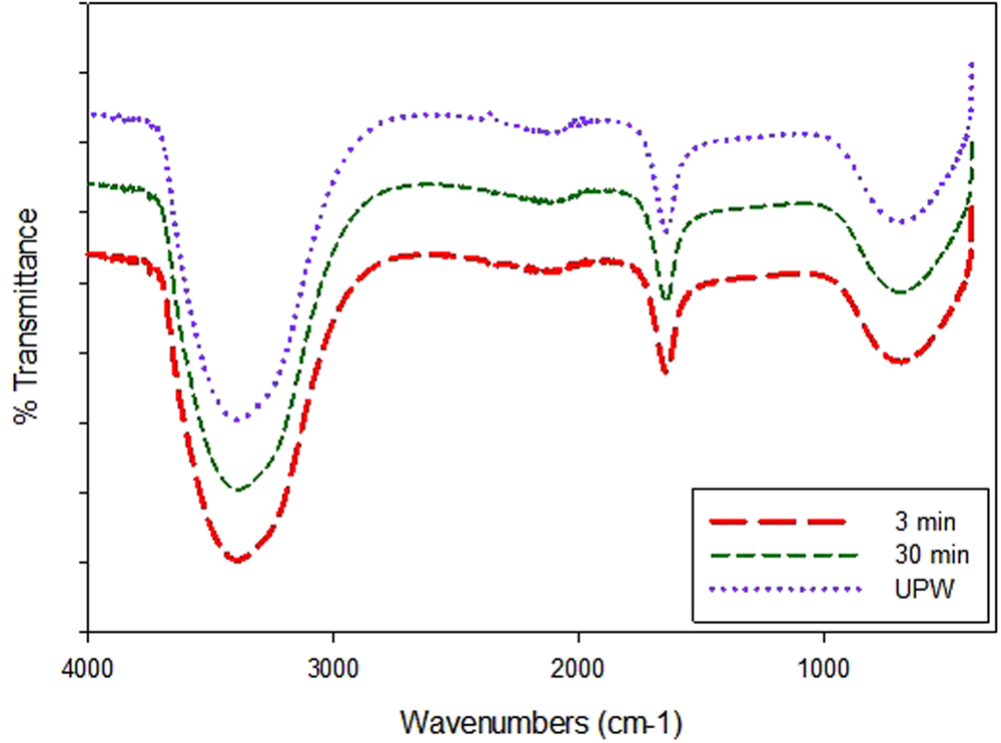
FTIR of ultrapure water (UPW) water and collected permeate after 3 and 30 minutes of filtration using UPW through PES/AG membrane (1 wt.% in the casting solution).

Moreover DW was filtered through the PES/AG membrane for 4 h before incubating *E.coli* bacteria on the membrane surface. As seen in Fig. 6c, only few bacterial cells were found on the surface of the conditioned membrane sample. Thus, the conditioned PES/AG membranes preserve their antifouling properties and this is indirect confirmation of the steady incorporation of AG into the membrane. The stability of AG compounds in the PES matrix was also assessed by recording of FTIR spectra of the permeate during filtration of DI water. As seen in Fig. 7 the permeate spectra collected after different filtration time were identical to spectra for DI water which indicates the absence of AG in the permeate and prove the stability of AG anchoring in the polymer matrix. Furthermore, the stability of the PS/AG membranes was also tested by ultra-sonication of the membrane samples for 30 min. Both the immersing water and the permeate collected after the filtration of ultrasonicated membranes with DI were analysed. The observed spectra were identical to that of DI, that further indicates the stability of the prepared membranes.

### Fouling test of the membranes with BSA

**Figure 8:**
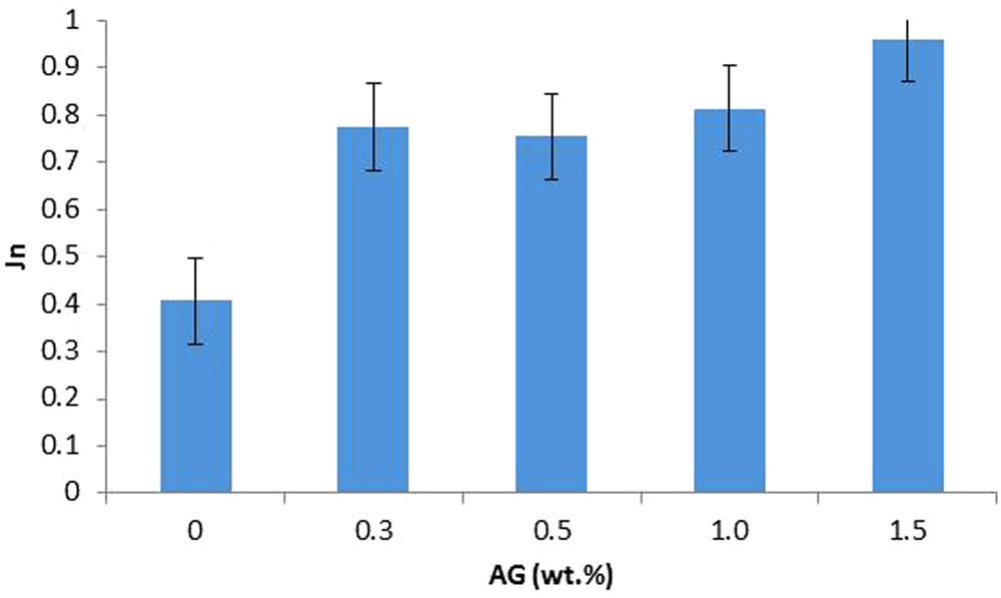
Normalized flux of the PES membranes containing 0, 0.3, 0.5, 1.0 and 1.5 wt.% AG in the dope solution after filtration of BSA solution (100 ppm) for two hours at pH 6.5.

The fouling test of the PES/AG membranes was conducted by filtering the BSA solution using dead end cell. Figure 8 shows the normalized water flux of PES/AG membranes with 0, 0.3, 0.5, 1.0 and 1.5 wt.% AG content in the dope solution after BSA filtration for two hours. Due to the increase in the hydrophilicity and surface charge, the AG-containing membranes showed higher normalized flux values when compared with pure PES membrane. The reduction in hydrophobicity and increase in the surface charge of the membrane has been reported by several researchers to lower the susceptibility of fouling due to the reduction in the interaction between the foulants and the membrane surface^66^.

### Mechanical testing of the membrane samples

**Figure 9:**
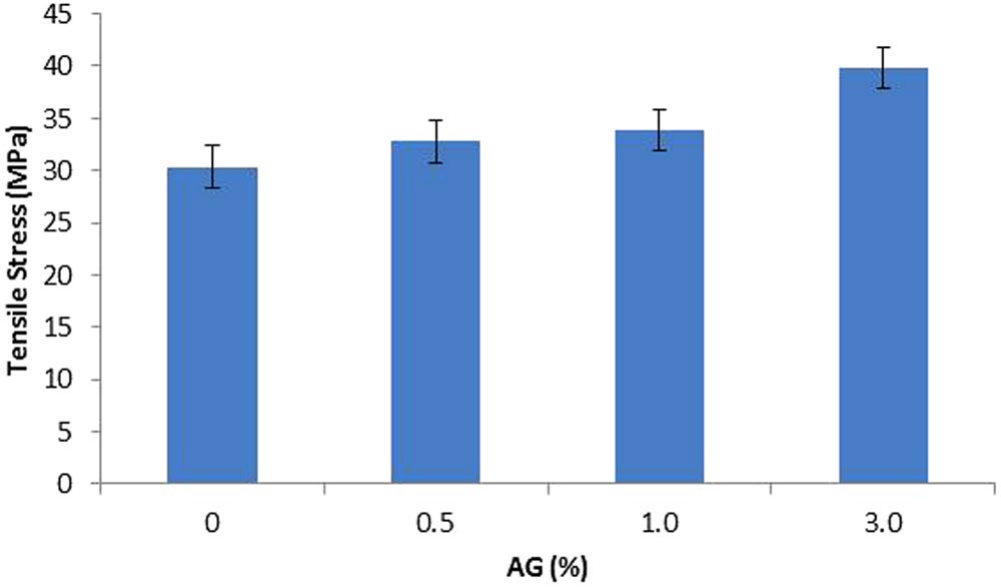
Stress values (MPa) of PES/AG membranes cast at different AG loading in the casting solution.

The mechanical properties of the fabricated membranes were characterized using tensile strength test with Brookfield CT3 Texture Analyzer (USA). The maximum tensile stress of PES/AG membranes achieved before snapping is shown in Fig. 9. It was seen that AG addition was found to enhance the mechanical properties of the membrane samples. For instance, PES membrane cast at 3 wt.% of AG demonstrated the increase in the tensile stress by about 31% compared to pure PES membranes. The possible explanation behind the significant enhancement in the mechanical properties of PES/AG membranes is that the addition of the amphiphilic high molecular weight AG to the casting solution results in the anchoring of AG macromolecules to the porous polymer PES matrix by means of the multipoint hydrophobic interactions between PES backbone and hydrophobic residues in AG macromolecules; this will in turn strengthen the porous membrane structure and increase its mechanical properties.

## Conclusions

In summary, in this work, AG was used for the first time as a new additive for the casting of PES membranes. The use of AG was found to remarkably enhance the surface charge, hydrophilicity, rejection, flux and antifouling properties of the fabricated membranes. The flux and the porosity of the PES/1wt.% AG membrane were increased by about 130% and 77%, respectively compared to pure PES membrane. The negative surface charge of the PES/3 wt.% AG membrane was increased from −11.1 to −24.6 mV while the contact angle of the same membrane was reduced by about 20% when compared to bare PES membranes. The reason behind the enhancement in the properties of the fabricated membranes can be attributed to the chemical composition of the AG macromolecules which contains a large number of hydrophilic residues, including carboxyl groups which attach themselves into the surface and the pore walls of the fabricated membranes. The overall effect of the increase in the surface charge and hydrophilicity of the membranes is the reduction in the membrane fouling with BSA. The bacterial tests showed that there was no growth of *E.coli* bacteria on the PES/AG membranes surface which means the fabricated membranes possess antibacterial properties. Moreover, the fabricated PES/AG membranes are capable to chelation with lead ions to enhance the rejection of the heavy metal. This study showed that AG is a promising additive to the casting solutions during fabrication of PES membranes with enhanced performance in water treatment applications.
